# Presence of two alternative *kdr*-like mutations, L1014F and L1014S, and a novel mutation, V1010L, in the voltage gated Na^+ ^channel of *Anopheles culicifacies *from Orissa, India

**DOI:** 10.1186/1475-2875-9-146

**Published:** 2010-05-28

**Authors:** Om P Singh, Cherry L Dykes, Manoj K Das, Sabyasachi Pradhan, Rajendra M Bhatt, Om P Agrawal, Tridibes Adak

**Affiliations:** 1 National Institute of Malaria Research, Sector 8, Dwarka, Delhi-110077, India; 2 National Institute of Malaria Research, Field Unit, TB Sanatorium Complex, Itki, Ranchi-835301, Jharkhand, India; 3 National Institute of Malaria Research, Field Unit, RLTRI Campus, Raipur-492015, India; 4 School of Studies in Zoology, Jiwaji University, Gwalior-474011, India

## Abstract

**Background:**

Knockdown resistance in insects resulting from mutation(s) in the voltage gated Na^+ ^channel (VGSC) is one of the mechanisms of resistance against DDT and pyrethroids. Recently a point mutation leading to Leu-to-Phe substitution in the VGSC at residue 1014, a most common *kdr *mutation in insects, was reported in *Anopheles culicifacies*-a major malaria vector in the Indian subcontinent. This study reports the presence of two additional amino acid substitutions in the VGSC of an *An. culicifacies *population from Malkangiri district of Orissa, India.

**Methods:**

*Anopheles culicifacies sensu lato (s.l.) *samples, collected from a population of Malkangiri district of Orissa (India), were sequenced for part of the second transmembrane segment of VGSC and analyzed for the presence of non-synonymous mutations. A new primer introduced restriction analysis-PCR (PIRA-PCR) was developed for the detection of the new mutation L1014S. The *An. culicifacies *population was genotyped for the presence of L1014F substitution by an amplification refractory mutation system (ARMS) and for L1014S substitutions by using a new PIRA-PCR developed in this study. The results were validated through DNA sequencing.

**Results:**

DNA sequencing of *An. culicifacies *individuals collected from district Malkangiri revealed the presence of three amino acid substitutions in the IIS6 transmembrane segments of VGSC, each one resulting from a single point mutation. Two alternative point mutations, 3042A>T transversion or 3041T>C transition, were found at residue L1014 leading to Leu (TTA)-to-Phe (TTT) or -Ser (TCA) changes, respectively. A third and novel substitution, Val (GTG)-to-Leu (TTG or CTG), was identified at residue V1010 resulting from either of the two transversions–3028G>T or 3028G>C. The L1014S substitution co-existed with V1010L in all the samples analyzed irrespective of the type of point mutation associated with the latter. The PIRA-PCR strategy developed for the identification of the new mutation L1014S was found specific as evident from DNA sequencing results of respective samples. Since L1014S was found tightly linked to V1010L, no separate assay was developed for the latter mutation. Screening of population using PIRA-PCR assays for 1014S and ARMS for 1014F alleles revealed the presence of all the three amino acid substitutions in low frequency.

**Conclusions:**

This is the first report of the presence of L1014S (homologous to the *kdr-e *in *An. gambiae*) and a novel mutation V1010L (resulting from G-to-T or -C transversions) in the VGSC of *An. culicifacies *in addition to the previously described mutation L1014F. The V1010L substitution was tightly linked to L1014S substitution. A new PIRA-PCR strategy was developed for the detection of L1014S mutation and the linked V1010L mutation.

## Background

*Anopheles culicifacies s.l. *is the most important malaria vector in the Indian subcontinent, affecting mainly rural areas [1]. This vector has developed widespread resistance against all the previously used insecticides such as DDT, dieldrin and malathion [2] and is developing resistance to pyrethroids [3]--the preferred group of insecticides for indoor residual spraying (IRS) and the only insecticide-class recommended for the impregnation of bed nets due to their relatively low mammalian toxicity and rapid knockdown effect on insects [4]. DDT and pyrethroids are neurotoxins which act on the voltage gated Na^+ ^channel (VGSC) leading to paralysis (knockdown) and eventual death of the insect. Knockdown resistance (*kdr*) due to reduced target site sensitivity is one of the mechanisms of resistance against these insecticides resulting from mutation(s) in the VGSC. The most common mutation known to be associated with knockdown resistance in insects is found at residue 1014 leading to Leu-to-Phe mutation [5] commonly referred to as *kdr *mutation. In *Anopheles gambiae*, two alternative *kdr *mutations L1014F [6] and L1014S [7] have been reported associated with knockdown resistance, which are referred to as *West African kdr *(*kdr-w*) and *East African kdr *(*kdr-e*), respectively. A variant mutation L1014C has been reported in *Anopheles sinensis *[8]. Recently a *kdr*-like mutation L1014F has been reported in the Indian malaria vector *An. culicifacies *from Surat district of Gujarat, India [9], which is resistant to both DDT and pyrethroids [3]. This study reports the presence of two additional amino acid substitutions present in the VGSC of an *An. culicifacies *population from Malkangiri district of Orissa, India, one of which is homologous to *kdr-e *of *An. gambiae *(L1014S) and the other a novel amino acid substitution V1010L resulting from two alternative point mutations.

## Methods

### Mosquito collection

Adult female *An. culicifacies *mosquitoes were collected from cattle sheds and human dwellings in the villages Hathiamba (block Kudumuluguma) and Takeguda (block Korukonda) of district Malkangiri of Orissa, India, in the year 2009. The mosquitoes were picked with the help of a mouth aspirator between 0600 and 0800 AM and transferred into a thermocol-box having a netted top. A water-soaked cotton pad was placed on the net to maintain adequate humidity. The mosquitoes were transported to the field-laboratory and were identified using key by Christophers [10]. Some of the fully blood-fed female *An. culicifacies *were allowed to attain semi-gravid stage and ovaries were pulled out and preserved in modified Carnoy's fixative (1:3 glacial acetic acid:ethanol). The mosquitoes (carcasses/intact mosquitoes) were preserved individually in isopropanol for further molecular analyses.

### DNA isolation and sequencing

DNA from individual mosquitoes was isolated using a method described by Livak [11]. A part of the IIS4-IIS5 linker-to-IIS6 segments of VGSC, encompassing at least 5 prospective residues reported to confer *kdr *resistance in insects [12], was amplified using two separate PCRs (hereafter referred to as 'PCR-I' and 'PCR-II') leaving a large intervening intron (1 kb). The primers used for the amplification were Kdr1F (5'-CTG AAT TTA CTC ATT TCC ATC A-3') and Kdr2R (5'-TTG AAA GCC ACG TAC CAT AAC A-3') for PCR-I, and KdrF (5'-GGA CCA YGA TTT GCC AAG ATG-3') and KdrR (5'-CGA AAT TGG ACA AAA GCA AAG-3') for PCR-II. The PCR conditions were same for both the PCRs comprising an initial denaturation at 95°C for 5 min, followed by 35 cycles, each at 95°C for 30 S, 48°C for 30 S and 72°C for 45 S, and a final extension step at 72°C for 7 min. The reaction mixture (25 μl) contained 1× buffer, 200 μM of each dNTP, 1.5 mM of MgCl_2_, 0.25 μM of each of the primers and 0.625 unit of AmpliTaq Gold taq polymerase (Applied Biosystems). The PCR products were purified by Qiaquick PCR purification kit (Qiagen) and sequenced using BigDye Terminator Kit v3.1 (Applied Biosystems) at in-house DNA sequencing facility or were sequenced by Macrogen Inc., South Korea. Most of the samples were sequenced from one direction only using primers Kdr2R in PCR-I and KdrF in PCR-II in order to save cost on sequencing. The sequences which showed ambiguity were sequenced from both directions. Initially, 20 samples were amplified and sequenced using PCR-I and PCR-II with an objective to screen the presence of non-synonymous mutations. Subsequently, more samples were sequenced using PCR-II in order to validate the genotyping data generated by PCR-based methods. During the entire study, a total of 112 samples were attempted sequencing using PCR-II, which includes 80 samples representing a single population (collected from a single locality in a day) alongside all the samples which were found positive for 1014S or 1014F by the PCR-based methods employed in this study.

### Genotyping of mosquitoes

#### Genotyping of L1014S

**Figure 1:**
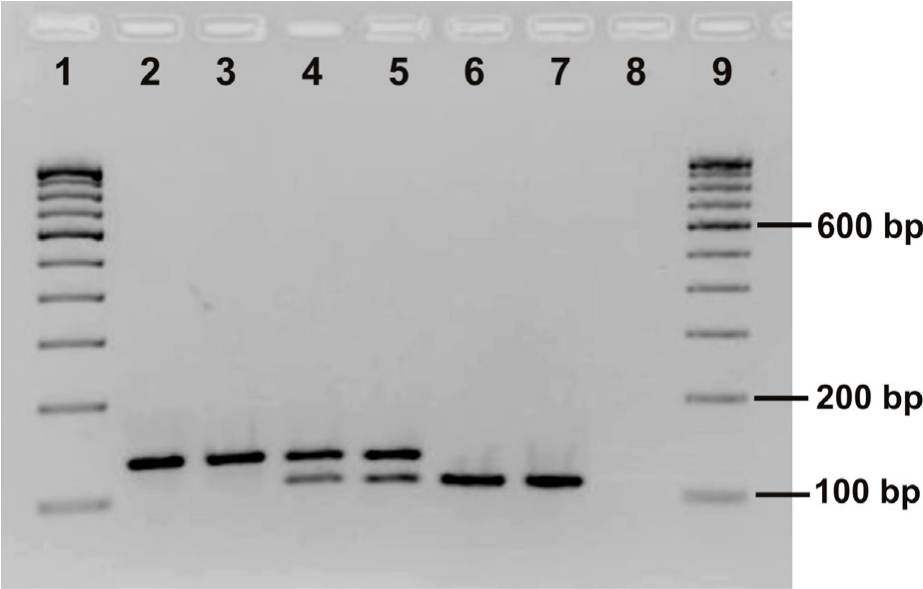
**Gel photograph showing result of PIRA-PCR for L1014S detection**. Lanes 1 & 9: 100 bp DNA ladder; lanes 2 & 3: homozygous wild type; lanes 4 & 5: heterozygotes; lanes 6 & 7: cloned PCR products with 1014S allele, isolated from heterozygous (1014L/S) samples; lane 8: negative control, without DNA.

For genotyping of L1014S mutation, a PIRA-PCR (hereafter referred to as 'PIRA-S') was developed. A primer KdrP1 (forward, 5'-TCC TGG CTA CAG TAT TGA TAG GgA ATT-3') was designed in which a deliberate mismatch (A-to-G, shown in small letter) was incorporated at the 5^th ^base from the 3' terminus; this creates a recognition site for restriction enzyme *EcoR*I (recognition site G|AATT|C) in the PCR amplicon, when amplified with a reverse primer KdrR, in case the 1014S (TCA) allele is present in the template DNA. No restriction site is formed in the presence of alternative alleles, i.e., 1014L or 1014F. The expected size of PCR amplified products is 130 bp and the size of cleaved products after treatment with *EcoR*I is 103 bp and 23 bp (excluding four bases 5'-overhang in each fragments). The 23 bp is unlikely to be detected on a 2.5% agarose gel. Therefore, presence of 103 bp cleaved product was scored as 1014S allele whereas presence of un-cleaved product (130 bp) was indicative of the presence of alternative alleles-1014L or 1014F. Presence of 103 bp alone was taken as criterion for scoring homozygous L1014S. For PIRA-S, the PCR products were amplified using 1.0 μM of KdrP1 and 0.5 μM of reverse primer KdrR in a reaction mixture (15 μL) containing 1× buffer, 200 μM of each dNTP, 1.5 mM of MgCl_2 _and 0.375 unit of AmpliTaq Gold taq polymerase (Applied Biosystems). The conditions of PCR were: initial denaturation at 95°C for 5 min, followed by 35 cycles of each at 95°C for 30 S, 48°C for 30 S and 72°C for 45 S, and a final extension step at 72°C for 7 min. Five μL of each PCR product were incubated with 5 units of *EcoR*I restriction enzyme (Fermentas Life Sciences) in a final volume of 20 μl at 37°C for 4 hours or overnight. The restriction products were run on 2.5% agarose gel containing ethidium bromide and visualized under UV illumination (Figure 1).

#### Genotyping of L1014F

For genotyping of L1014F mutation, an ARMS strategy by Singh *et al *[9] (hereafter referred to as 'ARMS-F') was adopted. Due to discovery of the presence of an alternative allele (1014S) at the same locus in *An. culicifacies *population, the original criterion of scoring of alleles was slightly modified, where presence of 191 bp band was taken as criterion for the presence of 1014L, 1014S or both instead of 1014L only.

### Cloning and sequencing

A total of seven samples from a population (population size = 80; collected from a single locality on a single day) which were found to be multi-site heterozygous after direct sequencing in respect to the amino acid positions 1010 and 1014, were amplified using PCR-II. The PCR products were purified using Qiaquick PCR Purification Kit, subsequently cloned in pGEM-T Vector System (Promega) according to the manufacturer's protocol and transformed into chemically competent *E. coli *DH5α. The transformants were plated on LB-Agar supplemented with 100 μg/ml ampicillin. Plasmid DNA was isolated from at least 10 white colonies originating from each mosquito sample by boiling them in 50 μl of TE buffer and subjected to PIRA-S and ARMS-F assays. Two clones from each mosquito sample, one positive for 1014S allele and the other for either 1014L or 1014F allele, were amplified using primers KdrF and KdrR, purified with Qiaquick PCR Purification Kit and sent to Macrogen, South Korea, for DNA-sequencing. During analysis any mutation found in the cloned product which was absent during direct sequencing was considered as PCR-error and excluded from the analysis.

### Hardy-Weinberg equilibrium analysis

The Exact-Test of Hardy-Weinberg equilibrium (HWE) of alleles was performed using the software Arlequin ver 3.11 [13].

### Linkage disequilibrium analysis

Analysis of linkage disequilibrium (LD) between alleles was performed using phased haplotype data of 79 samples from a population (n = 80), which were successfully sequenced using PCR-II. Haplotype assignment for all homozygotes and heterozygotes for single locus was done on the basis of direct sequences obtained. Phased haplotype sequence data for multi-site heterozygotes at amino acid positions 1010 and 1014 (as revealed by direct sequencing) were obtained after sequencing cloned products. Analysis of LD was done using the software DnaSP ver 5.10[14].

### Sibling species identification

For identification of sibling species, squash preparation of ovarian polytene chromosome was made following Green and Hunt [15] and examined under microscope using oil immersion lens (100×). Sibling species were identified on the basis of diagnostic inversion genotypes present on the X-chromosome and chromosome arm 2 following Subbarao *et al *[16].

## Results

### DNA sequence analysis

A total of 20 samples were sequenced for IIS4-S5 linker-to-IIS5 segments of VGSC using PCR product amplified with PCR-I, where no non-synonymous mutation is recorded.

DNA sequencing of a total of 111 specimens of *An. culicifacies s.l. *for IIS6 segment of VGSC (amplified with PCR-II) was successful and revealed the presence of two alternative non-synonymous mutations at residue L1014 leading to amino acid substitutions Leu (TTA)-to-Phe (TTT) or -Ser (TCA) resulting from 3042A>T transversion or 3041T>C transition, respectively, and a novel mutation at residue V1010 leading to Val (GTG)-to-Leu (TTG or CTG) substitution resulting from either 3028G>T or 3028G>C transversions. The numbers of various genotypes recorded after sequencing are shown in Table 1. Sequence analysis also revealed that all the 19 samples with the allele 1014S were also having allele 1010L (17 samples with 3028G>T and two samples with 3028G>C transversion). Few synonymous point mutations, all in heterozygous condition, were recorded which were: 2964A>G (n = 1), 3016C>T (n = 4), 3027A>T (n = 1) and 3045C>A (n = 1).

**Table 1:** Genotyping results of *An. culicifacies *samples by PCR-based assays and allelic association between L1014 and V1010 mutations as revealed by DNA sequencing

			L1014						
Genotyping methods	Genotypes								
			L/L	L/F	L/S	F/S	F/F	S/S	Total
**PCR-based assays**			176 (0.786)	26 (0.116)	18 (0.080)	2 (0.009)	2 (0.009)	0 (0.000)	224

		**V/V**	64	26	0	0	2	0	92
		
		**V/L**	0	0	17*	2	0	0	19*
**DNA-sequencing**	**V1010**								
		**L/L**	0	0	0	0	0	0	0
		
		**Total**	64	26	17	2	2	0	111

Figures in parenthesis indicate genotype frequencies. Letters L, F, S and V refer to leucine, phenylalanine, serine and valine amino acids, respectively.

*In two samples V1010L substitution was due to 3028G>C transversion and in rest samples due to 3028G>T transversion.

### Genotyping results

The results of genotyping of a total of 224 samples for 1014L, 1014F and 1014S alleles as determined by ARMS-F and PIRA-S are shown in Table 1. The allele frequencies of 1014L, 1014F and 1014S, based on PCR-based assays results, were 0.884, 0.071 and 0.045 respectively. The allele frequency of 1010L was not calculated as genotyping of this allele is based on DNA sequencing of selective samples. However, considering the fact that this allele was always found with 1014S in this study, it is presumed that the frequency of 1010L will be same as that of 1014S.

The various genotypes, as revealed by PCR-based assays, were in agreement with HWE (H_*O *_= 0.20536, H_*E *_= 0.21205, *p *= 0.58537). Results of PCR-based assays were well in agreement with the DNA-sequencing results of 111 samples sequenced successfully (Table 1). One sample with failed DNA sequencing was genotyped as 1014L/S by PCR-based assays.

### Allelic association of 1010L and 1014S

It was observed that one of the two alternative mutant alleles at V1010 residue, i.e., 3028G or 3028G>C (both leading to V1010L) and mutant allele 3041T>C (1014S) were found in the same individual. Cloning of seven samples with 1014S (in heterozygous condition) revealed that either of the two alternative mutant alleles 3028G>T or 3028G>C (1010L) and mutant allele 3041T>C (1014S) were located on the same haplotype. The wild allele 3028G (1010V) was found on haplotype with either 1014L (TTA) or 1014F (TTT). Linkage disequilibrium analysis using phased data derived from 79 individuals which were sequenced successfully revealed that the point mutations 3028G>T (V1010L) and 3041T>C (L1014S) are tightly linked (D' = 1.000, chi-square = 158.0, *p *< 0.001). One sample with 3028G>C mutation was not tested for LD, however, in this sample 3028G>C and 3041T>C were found on same haplotype.

### Sibling species composition

Out of a total of 50 samples examined for ovarian polytene chromosomes, 48 were successfully genotyped for the species-specific inversions. Forty-three (90%) samples were identified as species B and five (10%) as species C. Genotyping of these samples revealed that four of the species B and one of the species C were 1014L/1014F heterozygotes. Three of the species B and two of the species C were heterozygous for 1014L/1014S. Remaining samples were homozygous 1014L. Analyses of the data with Fisher's exact test revealed absence of association of any of the two mutations with any sibling species (*p *> 0.05 for both the mutations).

## Discussion

Knockdown resistance resulting from target site insensitivity in insects is one of the mechanisms of resistance against DDT and pyrethroids which is conferred by amino acid substitution(s) in the VGSC. In anophelines, the most commonly reported mutation conferring knockdown resistance is at residue L1014 leading to Leu-to-Phe substitution, often referred to as *kdr *mutation and has been reported in several anophelines such as *An. gambiae *[6], *Anopheles arabiensis *[17], *Anopheles stephensi *[18], *Anopheles subpictus *[19], *An. sinensis *[8], *Anopheles sacharovi *[20] and *An. culicifacies *[9]. Variant mutations, Leu-to-Ser and Leu-to-Cys, at this residue have been reported in *An. gambiae *[7] and *An. sinensis *[8], respectively. In *An. culicifacies*, only one point mutation (A-to-T transversion) leading to Leu-to-Phe amino-acid substitution at position 1014 was reported from Surat district of Gujarat, west India, which is resistant to both DDT and pyrethroids [9]. This study revealed the presence of three other non-synonymous point mutations in *An. culicifacies*, two at position 1010 (G-to-T or -C), each one leading to Val-to-Leu substitution, and one T-to-C transition at position 1014 leading to Leu-to-Ser substitution in a population from district Malkangiri of Orissa. The two alternative mutations L1014F and L1014S are homologous to mutations found in *An. gambiae*, referred to as *kdr-w *and *kdr-e*, respectively. The V1010L substitution resulting from either of the two alternative point mutations is a novel mutation and was found linked with L1014S irrespective of the types of associated point mutations. Cloning experiment and LD analysis showed that these two amino acid substitutions L1014S and V1010L are found on the same haplotype and not independently.

The role of L1014F and L1014S mutations in conferring DDT/pyrethroid resistance has already been established in many insects including *An. gambiae *[7,21-24]. Their role in *An. culicifacies *is uncertain, however, the conserved nature of these mutations particularly that of the classic mutation L1014F in several insects [25] suggests a similar role in other species. The role of novel mutation V1010L in knockdown resistance is unknown. However, the presence of two alternative point mutations responsible for a same amino acid substitution (V1010L) in a population indicates that this amino acid substitution has been favoured during evolution probably due to their role in protection against knockdown. Further, the fact that V1010L (3028G>T or 3028G>C) always co-existed with L1014S, suggests that the V1010L substitution may probably have complementary role in knockdown resistance in association with L1014S. Their independent role in knockdown resistance is difficult to establish in the population studied because they are found to exist together. Although limited data from one geographic region suggest the absence of recombination, screening of larger population from different geographical localities may reveal recombination and these two alleles may be found independently.

The frequency of *kdr*-like mutations L1014F and L1014S (and linked V1010L) is very low in the population studied and these alleles were found mostly in heterozygous conditions with less than 1% homozygotes. The alleles were, however, well in agreement with HWE. This is contrary to a report by Hoti et al [26] carried out in the same area, where the frequency of homozygous RR (1014F) was too high (71%) as compared to heterozygotes (4%), resulting in significant departure from HWE (*p *= 0.00000, Exact-test) due to deficiency of heterozygotes. One possible reason for this departure may be genotyping error due to severe mismatch in flanking primers, which were basically designed for *An. gambiae *[9]. Changes in gene frequency over time due to changes in sibling species composition or insecticide pressure may be other possible reasons that can account for the difference in allele frequency.

The *An. culicifacies *population in Malkangiri consists mainly species B with low proportion of species C. Preliminary analysis based on limited numbers of samples could not establish any association of any of the *kdr*-like mutations with sibling species. Since the analysis presented in this study is based on a small sample size, particularly in the case of species C (n = 5), it is emphasized that larger number of samples should be analyzed to establish an association of particular *kdr*-like mutations with sibling species.

A new PIRA-PCR assay (PIRA-S) was developed for genotyping of L1014S mutation and the results obtained through this assay were in agreement with direct sequencing results. PIRA-PCR was preferred to ARMS for genotyping of L1014S because the ARMS assay tried for this mutation resulted in poor amplification whereas PIRA-PCR provided discrete and better yield of the amplified product. The PIRA-S is very cost effective as the restriction enzyme *EcoR*I selected for this PIRA-PCR is inexpensive (0.5 cent/unit). No assay was developed for the novel mutation V1010L because it always co-existed with L1014S.

The *kdr *factor is reportedly recessive [27] or incompletely recessive [22], therefore, the effect of *kdr *on phenotypic resistance can best be studied in a population where sufficient number of homozygous individuals for *kdr*-like alleles are present. In this study area, the frequency of homozygous mutant alleles is extremely low (<1%) and, therefore, it will be difficult to establish the phenotypic effect of these mutations in this population. Authors are attempting to colonize *An. culicifacies *having different *kdr*-like mutations to establish their role in phenotypic knockdown resistance.

## Conclusions

This is the first report of presence of L1014S mutation (homologous to *kdr-e *in *An. gambiae*) and a novel mutation V1010L (resulting from two alternative transversions) in the VGSC of *An. culicifacies *in addition to previously described mutation L1014F. The V1010L is tightly linked with L1014S irrespective of the type of point mutations associated with latter. A new and specific PIRA-PCR strategy was developed for the detection of L1014S mutation and the linked V1010L mutation.

## Competing interests

The authors declare that they have no competing interests.

## Authors' contributions

OPS designed the study, analysed sequences, designed PIRA-PCR strategy, performed statistical analyses and wrote the manuscript; CLD performed genotyping, SP did cloning experiments, MKD and RMB organized field work, OPA and TA contributed to the manuscript. All authors provided critical reviews of the manuscript and approved the final version.
